# The potential of essential oils as mosquito (Diptera: Culicidae) bio-repellents: a systematic review

**DOI:** 10.1590/S1678-9946202668039

**Published:** 2026-07-03

**Authors:** Adeilson Pimentel Rabelo, Rodrigo Alves Soares Cruz, Everson dos Santos David, Erique da Costa Fonseca, Karen Carmo dos Santos, Raimundo Nonato Picanço Souto

**Affiliations:** 1 Universidade Federal do Amapá, Laboratório de Arthropoda, Macapá, Amapá, Brazil; 2 Universidade Federal do Amapá, Programa de Pós-Graduação em Ciências Farmacêuticas, Macapá, Amapá, Brazil; 3 Universidade Federal do Amapá, Laboratório de Nanotecnologia Fitofarmacêutica, Macapá, Amapá, Brazil; 4 Universidade Federal do Amapá, Programa de Pós-Graduação em Biodiversidade e Biotecnologia na Amazônia Legal (Rede Bionorte), Macapá, Amapá, Brazil

**Keywords:** Repellents, Essential oils, Culicidae, Pathogens

## Abstract

Controlling culicid-borne diseases remains a global public health challenge. While public health actions are vital, individual protection via repellents is essential. Concerns regarding synthetic compounds such as DEET have intensified the search for sustainable plant-derived alternatives. This systematic review analyzed 33 experimental studies that were published from 2020 to 2024. A comprehensive search was performed on SciELO, PubMed, Scopus, Web of Science, ScienceDirect, and LILACS using the keywords "Repellency," "Essential Oils," and "Mosquitoes." Data were independently selected and extracted to evaluate protection time and repellency percentages. The included studies showed low risk of methodological bias according to SYRCLE. Results showed that most of the tested essential oils possess high protective potential. Species such as *Perilla frutescens, Leucas stachydiformis*, *Cymbopogon nardus*, and *Eucalyptus camaldulensis* achieved up to 100% repellency, with protection exceeding eight hours. The cage test was the predominant laboratory method. Essential oils are promising, effective alternatives to synthetic repellents, offering reduced environmental and toxicological impacts. However, this evidence is based on controlled laboratory conditions rather than field trials. This limitation may affect real-world effectiveness, and further research in natural environments is recommended to validate these results for commercial application.

## INTRODUCTION

Mosquito control configures a global public health challenge due to their role as vectors of arboviruses and parasites that cause disease, such as dengue, yellow fever, zika, chikungunya, filariasis, and malaria, which drives the growing search for effective and safe control methods^1^. These pathogens affect millions of people worldwide, significantly impacting tropical and subtropical regions^1^.

Traditional strategies for controlling mosquito vectors primarily rely on integrated management approaches. These commonly include the use of chemical larvicides and adulticides, the distribution of insecticide-treated bed nets, and environmental management that aim to eliminate breeding sites to reduce vector populations^2,3^.

Although actions by public health agencies are fundamental, individual protection via personal repellents is essential to reduce the frequency of bites and the risk of infection^3^. However, the continuous use of synthetic repellents, such as DEET (N,N-diethyl-3-methylbenzamide) and Icaridin, has raised concerns due to their potential adverse effects, including skin irritation, neurotoxicity, and environmental impact^4,5^. Despite their widespread use, these traditional methods face growing challenges, such as the emergence of insecticide resistance and the need for continuous community engagement^2^ hence the increasing interest in sustainable and eco-friendly alternatives, particularly plant-derived essential oils, which offer a promising path for safe and effective mosquito control^6^.

These oils show bioactive properties, including antimicrobial, antioxidant, and, notably, repellent activities^5^. Oils from species such as *Corymbia citriodora* (Hook.), citronella (*Cymbopogon* spp.), and andiroba (*Carapa guianensis* Aubl.) have been widely studied for their ability to repel mosquitoes such *Aedes aegypti* (the primary vector of dengue, zika, and chikungunya viruses) and *Anopheles* spp. (responsible for transmitting *Plasmodium* parasites that cause malaria)^7,8^.

This systematic review aims to investigate the scientific literature for evidence on the efficacy of essential oils as repellents to prevent contact with vector mosquitoes, focusing on safe and effective alternatives for individual protection.

## MATERIALS AND METHODS

### Protocol design and registration

This review sought to answer the guiding question: "What is the evidence for the efficacy of essential oil-based repellency tests against mosquitoes in humans?" For this end, this study was designed according to the Preferred Reporting Items for Systematic Reviews (PRISMA) 2020^9^ and submitted to the International Prospective Register of Systematic Reviews for methodological analysis under registration Nº CRD42024613854.

### Source of information and search strategy

Searches were conducted on the following electronic databases: Scientific Electronic Library Online, US National Library of Medicine, Web of Science, Scopus, ScienceDirect, and LILACS. The Boolean operator "AND" was used between the terms "Repellency," "Essential Oils," and "Mosquitoes."

### Eligibility criteria

Studies were included in this review if they met the following criteria: (I) published in English, Portuguese, and Spanish; (II) studies that verified the repellent effect of essential oils on Culicidae; and (III) studies that reported efficacy using at least one of the following indicators: protection time (minutes/hours) or percentage of repellency. The following were excluded: systematic reviews, field studies with repellents, studies that used nanotechnology (e.g., nanoemulsions, emulsions), studies that used non-human animals in repellency tests, studies on repellents focusing on other organisms (such as ticks or agricultural fungicides), studies that used extracts in repellency tests, and studies prior to 2020. Moreover, studies from which it was impossible to extract information were also excluded. The screening of titles and abstracts was performed by three researchers (APR, ESD, and KCS), and the full text of the articles was read independently by two researchers (APR and ESD).

### Study selection and data extraction

The search results were exported to the Mendeley reference manager (version 1.18) for duplicate removal. Subsequently, the files were uploaded to Rayyan QCRI for study selection. Initially, titles and abstracts were screened by three independent researchers (APR, ESD, and KCS), with a third researcher (KCS) acting as an arbiter in cases of disagreement. Following this initial screening, the full texts were read independently by two authors (APR and ESD).

The extracted data were plotted onto Microsoft Excel 2019 spreadsheets. The categories for extraction included study type (laboratory), publication period (2020–2024), target population (humans or different mosquito species), repellent types (comparison between natural and synthetic), language, and efficacy assessment methods. Additionally, methodological quality parameters, such as clear sample definition, experiment replicability, and statistical analysis, were recorded. Any discrepancies during data synthesis were resolved by a third researcher (KCS).

### Risk of bias

Overall, two authors (APR and ESD) assessed the risk of bias using the systematic review centre for laboratory animal experimentation (SYRCLE) tool for animal studies, which is an adapted version of the Cochrane RoB tool^10^. The choice of this tool is justified by the methodological aspects related to the handling of mosquitoes (Culicidae), which constitute the animal model for the repellency assays. The tool includes domains such as selection, performance, detection, attrition, reporting, and other biases. Signaling questions are applied to the articles to aid in judgment: "Yes" indicates a low risk of bias, "No" indicates a high risk of bias, and "?" indicates an unclear risk of bias.

## RESULTS

### Study selection

**Figure 1:**
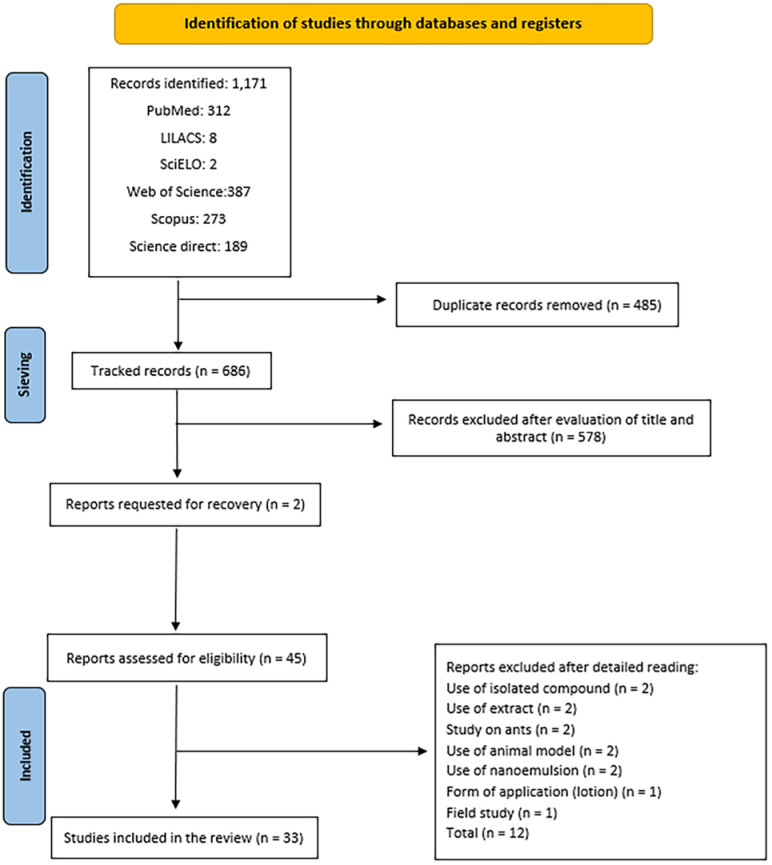
Flowchart of the Prisma 2020 repellent study selection.

The initial search retrieved 1,171 records from the six electronic databases. After removing duplicates and selecting titles and abstracts, this study evaluated 45 full-text articles for eligibility. Of these, 33 studies were considered eligible for inclusion in this review. The PRISMA flow diagram (Figure 1) details the complete selection process and reasons for exclusion.

Regarding efficacy parameters, 26 studies (78.8%) reported protection time and repellency percentage, whereas seven (21.2%), only one of these metrics. To ensure a comprehensive synthesis, eligibility criteria included studies that provided at least one of these primary outcomes.

### Characteristics of the included studies

This review only included studies that met the inclusion criteria for essential oil-based repellents against Culicidae. The selected studies were published from 2020 to 2024 and conducted in laboratories. Table 1 shows the characteristics and main results of the studies. Overall, the studies were conducted in several countries, with the following distribution: Pakistan (n=6; 18.2%), Thailand (n=4; 12.1%), China (n=3; 9.1%), India (n=3; 9.1%), Brazil (n=3; 9.1%), Indonesia (n=2; 6.1%), Vietnam (n=1; 3.0%), Ethiopia (n=1; 3.0%), Uganda (n=1; 3.0%), Ghana (n=1; 3.0%), Madagascar (n=1; 3.0%), Italy (n=1; 3.0%), Iran (n=1; 3.0%), Greece (n=1; 3.0%), Kenya (n=1; 3.0%), Malaysia (n=1; 3.0%), and one study fail to specify the location in which it was carried out (n=1; 3.0%).

**Table 1:** Stratification of the description of studies included in the systematic review on the potential of essential oils as mosquito (Diptera: Culicidae) bio-repellents

Article	Country	Plant	Volunteers	Concentrations	Mosquitoes	Repellency %	Time (min/ hr)
Parveen *et al*.^26^	Pakistan	*Artemisia scoparia*	-	33.3 μg/cm^2^ 333 μg/cm^2^	*Aedes aegypti*	100	75 min 60 min
Boonyuan *et al*.^19^	Thailand	*Cananga odorata* *Vetiveria zizanioides*	2	2,8 ml	*Culex quinquefasciatus*	91.67 80 88	-
Zhang *et al*.^1^	China	*Perilla frutescens*	-	5% 10%	*Culex pipiens pallens*	100	4 – 6 h
Fikru *et al*.^23^	Ethiopia	*Leucas stachydiformis*	2	366.7 μg/cm 133.3 μg/cm 41.6 μg/cm	*Anopheles arabiensis*	100	4 h
Batume *et al*.^40^	Uganda	*Nepeta cataria*	2	2% 100%	*Aedes aegypti*	85	1 h 5 h
Opoku-Bamfoh *et al*.^14^	Ghana	*Morinda citrifolia* *Moringa oleifera* *Ocimum basilicum*	3	20% 90%	*Anopheles Kisumu* and *Anopheles gambiae*	90	15 – 240 min
De Brito *et al*.^7^	Brazil	*Cymbopogon winterianus* *Eucalyptus globulus lippia alba* *Lippia thymoides*	-	1.25 mg/mL 2.5 mg/mL 5 mg/mL 10 mg/mL 20 mg/mL	*Aedes aegypti*	70	-
Haris *et al*.^13^	Pakistan	*Azadirachta indica* *Citrus reticulata* *Dysphania ambrosioides* *Eucalyptus camaldulensis* *Cymbopogon citratus* *Mentha longifolia* *Perovskia atriplicifolia* *Salvia moorcroftiana*		33 μg 165 μg 330 μg	*Aedes aegypti*	10 – 100	60 – 135 min
Haris *et al*.^24^	Pakistan	*Carpesium abrotanoides*	15	1% 5% 10%	*Aedes aegypti*	40	45 – 315 min
Dutta *et al*.^17^	India	*Aromatic homalomena* *Citrus macroptera*	-	1:1 1:2 1:3 2:1 3:1	*Aedes aegypti*	-	4 h 6 h
Benelli *et al*.^25^	Madagascar	*Hazomalania voyronii*	-	10% 50 % 100%	*Culex quinquefasciatus* and *Aedes aegypti*	20 35 80 80	30 – 120 min
Manh and Tuyet^5^	Vietnam	*Mentha arvensis*	2	25% 50% 100%	*Aedes aegypti*	-	45 –165 min
Mustapa *et al*.^11^	Indonesia	*Cinnamomum burmanii* *Myristica fragrans* *Cymbopogon citratus* *Pogostemon cablin* *Curcuma longa* *Cymbopogon nardus* *Syzygium aromaticum*	3	10%	*Aedes aegypti*	95.2 94.7 85 83.8 80.4 72.2 71.4	6 h
Kamaraj *et al*.^8^	India	*Citrus limon* *Cymbopogon nardus* *Eucalyptus globulus* *Mentha* *Trachyspermum ammi*	-	100 ppm	*Aedes aegypti* *Anopheles stephensi culex quinquefasciatus*	96 94 84	8 h
Tian *et al*.^27^	China	*Eucalyptus cloeziana, Eucalyptus umbellata* *Eucalyptus benthamii* *Eucalyptus globulus* *Melaleuca alternifolia*	3	12.5% 25.0% 50.0%	*Culex pipiens quinquefasciatus*	8.7 5.5 15.0	15 – 465 min
Deng *et al*.^22^	China	*Foeniculum vulgare* *Cinnamomum zeylanicum* *Rhizoma zingiberis* *Mentha haplocalyx* *Piper nigrum* *Pogostemon cablin* *Syzygium aromaticum*	1	020% 025% 2.50% 10% 20% 25% 30%	*Aedes albopictus* *Aedes aegypti*	50% 95% 99%	29 – 115 min
Najar *et al*.^28^	Italy	*Salvia dolomitica* Salvia *dorisiana* *Salvia sclarea* *Salvia somalensis*	1	0,02 nL cm ^−2^ 200 nL cm ^−2^	*Aedes albopictus*	38.68 39.88 12.65 83.54	5 – 60 min
Abbas *et al*.^4^	Pakistan	*Ailanthus altissima* *Erigeron bonariensis* *Erigeron canadenses* *Mentha longifolia* *Salsola imbricata* *Zanthoxylum armatum*	1	1% 5% 10%	*Aedes aegypti*	63 100 100 50 10 96.5 100	4.60 – 43.28 min
Sheikh *et al*.^2^	Iran	*Eucalyptus globulus e Syzygium Aromatum*	-	0.5% 1% 3% 5% 10%	*Anopheles stephensi*	83.33 88.89 94.44 88.89 0	15 min 30 min 0.75 h 30 min
Sutthanont *et al*.^12^	Thailand	*Cymbopogon nardus* *Citrus bergamia* *Coriandrum sativum* *Citrus aurantium* *Ocimum basilicum* *Pimpinella anisum* *Pogostemon cablin* *Mentha piperita* *Rosmarinus* *officinalis* *Salvia officinalis* *Vetiveria zizanioides*	-	100 µL Undiluted oil	*Aedes aegypti Anopheles dirus Culex quinquefasciatus*	-	30 – 330 min
Liakakou *et al*.^29^	Greece	*Pinus nigra* *Pinus heldreichii* *Cristo Pinus pinea* *Juniperus turbinata* *Juniperus oxycedrus deltoides*	-	0,2 μL/cm^2^	*Aedes albopictus*	41.8 42.0 36.1 82.4 22.3 100	2 h
Ndirangu *et al*.^30^	Kenya	*Nigella sativa*	-	10 g/mL 10-1 g/mL 10-2 g/mL 10-3 g/mL 10-4 g/mL	*Anopheles gambiae*	100 100	30 – 360 min
Oliveira *et al*.^36^	Brazil	*Origanum vulgare Thymus vulgaris*	-	0.01% 0.1% 0.5% 1.0% 2.5% 5.0%	*Aedes aegypti*	37.8 68.9	-
Ludia *et al*.^3^	Indonesia	*Ocimum basilicum*	-	10% 15% 20% 25%	*Aedes aegypti*	67,6 100	30 min
Iqbal *et al*.^16^	Pakistan	*Agrimonia eupatoria* *Lepidium pinnatifidum* *Mentha longifolia* *Origanum vulgare*	5	1% 5% 10%	*Aedes aegypti*	100 83 94	1 – 6h
Osei-Owusu *et al*.^31^	Ghana	*Ocimum* gratissimum *Ocimum* tenuiflorum *Ocimum basilicum*	5	50% 10% Undiluted oil	*Anopheles gambiae*	60 10 20 90 50 30 20	30 – 105 min
Jhaiaun *et al*.^37^	Thailand	*Psidium guajava*	-	-	*Anopheles minimus* *Anopheles epiroticus* *Culex quinquefasciatus*	100 98 91.67 44.23 35.00 11.00	30 – 90 min
Adhikari *et al*.^32^	India	*Citrus aurantifolia* *Citrus maxima* *Citrus aurantium* *Citrus limon* *Citrus medica* *Citrus paradise*	-	0.1 mg/cm ^2^ 0.2 mg/cm ^2^ 0.25 mg/cm ^2^ 0.5 mg/cm ^2^ 1 mg/cm ^2^ 2 mg/cm ^2^ 3 mg/cm ^2^ 4 mg/cm ^2^ 5 mg/cm ^2^ 6 mg/cm ^2^	*Aedes aegypti*	EC50 = 0.46 CEC50 = 0.67 EC50 = 0.79 CEC50 = 2.41 EC50 = 2.63 EC50 = 2.88 CE50 = 0.14	-
Wahab *et al*.^38^	Malaysia	*Citrus iners*	-	1% 5% 10%	*Aedes aegypti*	12.33 10.78 10.85	3 h
Silva *et al*.^39^	Brazil	*Blepharocalyx salicifolius* *Campomanesia adamantium* *Eugenia dysenterica* *Myrcia dictyophylla* *Protium ovatum* *Xylopia aromatic*	-	1%	*Culex quinquefasciatus*	71 61 60 35 42 44 93	30 min
Abbas *et al*.^33^	Pakistan	*Calista Callistemon viminalis Helichrysum odoratissimum Hyptis suaveolens* *Lantana camara Schinus tereSchinus terebinthifolia*		1% 10%	*Aedes aegypti, Anopheles gambiae* and *Culex quinquefasciatus*	90 52 5,6 100 100 90 52 100 50	-
Paulraj *et al*.^34^	Not specified	*Lavandula latifolia*	1	100 ppm 200 ppm 300 ppm 400 ppm 500 ppm	*Aedes aegypti*	100	30 –180 min
Sukkanon *et al*.^20^	Thailand	*Cananga odorata*	-	0.5% 1.0% 2.5% 5.0%	*Aedes aegypti Culex quinquefasciatus Anopheles dirus Anopheles minimus*	98.4 64.4 39.3	150 min

Concentration units vary between studies (such as %, µg/cm^2^, mg/mL, ppm, nL/cm^2^) reflecting the different methodological protocols and international guidelines followed by the authors. Unless explicitly identified as a "mixture" or ‘combination’ in the "Plant" column, each botanical species was tested separately in each study to assess its individual repellent potential. Essential oils were applied in their pure form or diluted in solvents (such as ethanol or acetone) according to the experimental design of each study.

### Data analysis and synthesis of evidence

The essential oils were stratified according to their repellency percentage, ranging from 10% to 100%.

The highest repellency percentages occurred in *Perilla frutescens, Leucas stachydiformis, Eucalyptus camaldulensis, Artemisia scoparia*, *Cymbopogon nardus, Lavandula latifolia*, and *Salvia moorcroftiana* essential oils, all showing 100% repellency. Concentrations of 5% and 10% of *Perilla frutescens* essential oil achieved 100% repellency against *Aedes aegypti*.

Regarding the essential oils with the longest protection times, *Leucas stachydiformis, Homalomena aromatica, Citrus macroptera, Eucalyptus benthamii*, and *Ocimum basilicum* L. were notable against several culicid species. Of the 33 chosen articles, 24 had data on protection time and repellency percentage, whereas seven only described their results on repellency percentages.

The following section describes the essential oils from plant species most frequently investigated as repellents (that is, in more than two studies).

### *Cymbopogon* spp (Citronella)

This research found that five studies investigated *Cymbopogon* spp. essential oils^2,7,8,11,12^, showing their prominence as candidates for natural repellents due to their high content of active compounds, such as citronellal, extracted from various species of this genus.

Brito *et al*.^7^ assessed the repellent activity of *Cymbopogon winterianus* essential oil against *Aedes aegypti*, with a protection rate of 70%. The authors also found citronella (22.8%) as one of the main constituents of the essential oil.

Haris *et al*.^13^ observed that the essential oil of *Cymbopogon citratus* provided 100% protection against female *Aedes aegypti*. Conversely, Mustapa *et al*.^11^ evaluated the repellent activity of essential oils from various plants, including *Cymbopogon nardus*, against *Aedes aegypti, Anopheles stephensi*, and *Culex quinquefasciatus*. At a concentration of 100 ppm, these oils showed high efficacy, with repellency rates ranging from 84% to 96% and providing protection for up to eight hours.

Kamaraj *et al*.^8^ also conducted studies with *Cymbopogon nardus* and other species against *Aedes aegypti, Anopheles stephensi*, and *Culex quinquefasciatus*, achieving repellency rates ranging from 84% to 96%, with a protection time of up to eight hours. Sutthanont *et al*.^12^ tested a 10% solution of *Cymbopogon nardus* against *Aedes aegypti, Anopheles dirus*, and *Culex quinquefasciatus*, recording protection time of 90, 180, and 360 min, respectively.

### *Eucalyptus* spp.

Investigations were carried out on *Eucalyptus* species. Brito *et al*.^7^ chose *Eucalyptus globulus* for repellency tests against *Aedes aegypti*, achieving a protection rate above 70%. Haris *et al*.^13^ studied the essential oils of *Eucalyptus camaldulensis* and other species, obtaining protection rates ranging from 10% to 100% depending on the concentration (33 to 330 μg), as detailed in Table 1.

Kamaraj *et al*.^8^ and Sheikh *et al*.^2^ investigated the repellent effect of *Eucalyptus globulus*, reporting repellency rates greater than 83%. However, protection times varied considerably between studies, with Kamaraj *et al*.^8^ recording up to eight hours (at 100 ppm), whereas Sheikh *et al*.^2^ observed shorter protection periods, ranging from 15 to 45 min, depending on the concentration.

### *Ocimum basilicum* (Basil)

*Ocimum basilicum,* a species of the Lamiaceae family, is known for its repellent activity. Opoku-Bamfoh *et al*.^14^ tested 20%, 40%, 60%, 80%, and 90% concentrations and obtained protection that ranged from 50% to 90% depending on the concentration and protection times of 84 to 105 min.

Sutthanont *et al*.^12^ reported the protection time against three culicid species (*Aedes aegypti, Anopheles dirus*, and *Culex quinquefasciatus*) using 100 µL of the undiluted essential oil. This study (carried out in Thailand) showed that the protection against *Aedes aegypti* totaled 180 min; *Anopheles dirus*, 90 min; and *Culex quinquefasciatus*, 360 min.

Ludia *et al*.^3^ observed similar results using 10%, 15%, 20%, and 25% essential oil concentrations in tests against *Aedes aegypti*. The concentrations showed a repellent effect of 98% to 100% in the first hour, which decreased to 67.6% and 81.3% after six hours.

### *Mentha* spp. (Mint)

The genus *Mentha* is of great importance within the Lamiaceae family, with wide distribution and cultivation in various countries. The pharmaceutical industry extensively uses its essential oil for medicinal purposes due to its safety and popularity among consumers^15^. This research found that five of the included studies reported the repellent effects of *Mentha* spp..

Abbas *et al*.^4^ found that the essential oil of *Mentha longifolia* showed 100% repellency immediately after application (comparable to DEET), an effect that lasted for up to two hours. Iqbal *et al*.^16^ and Haris *et al*.^13^ also observed a similar effect with the same species but with a protection time of up to 90 min.

The retrieved research also evaluated other species. Manh *et al*.^5^ investigated *Mentha arvensis* L. against *Aedes aegypti*, obtaining 45% to 100% protection at concentrations of 25%, 50%, and 100%. Furthermore, Kamaraj *et al.* ^8^ observed that 100 ppm of the *Mentha arvensis* L. essential oil provided 84% to 95% repellency for up to eight hours.

### *Citrus* spp. (Citrus fruits)

This research found that four of its studies investigated the repellent effects of *Citrus* spp., a genus that includes various citrus fruits.

In Haris *et al*.^13^, the essential oil of *Citrus reticulata* at concentrations of 33, 165, and 330 µg/cm^2^ showed 100% repellency against *Aedes aegypti*, with protection time ranging from 60 to 135 min. Another study using *Citrus macroptera* found the protection time ranged from four to six hours^17^.

Kamaraj *et al*.^8^ studied *Citrus limon* L., which provided up to eight hours of protection. Sutthanont *et al*.^12^ evaluated the repellent activity of two species, *Citrus bergamia* (bergamot) and *Citrus aurantium* (bitter orange), using 100 µL of the undiluted essential oils against three culicid species: *Aedes aegypti, Anopheles dirus*, and *Culex quinquefasciatus*.

The *Citrus bergamia* oil provided 90 min of protection against *Aedes aegypti* and *Anopheles dirus* and 220 min against *Culex quinquefasciatus*. Meanwhile, the *Citrus aurantium* oil offered 270 min of protection against *Aedes aegypti*, 180 min against *Anopheles dirus*, and 360 min against *Culex quinquefasciatus*.

### Cananga odorata

*Cananga odorata*, a tropical Asian plant from the Annonaceae family, is widely used in the cosmetics industry due to its pleasant aroma^18^. Its essential oil, at a volume of 2.8 mL, showed 80% repellency against *Culex quinquefasciatus* in non-contact and contact tests, as in Boonyuan *et al*.^19^.

In Thailand, Sukkanon *et al*.^20^ investigated excito-repellency in four culicid species (*Aedes aegypti, Culex quinquefasciatus, Anopheles dirus*, and *Anopheles minimus*), using 0.5%, 1.0%, 2.5%, and 5.0% essential oil concentrations. The authors considered repellency as promising, with rates above 50% for the tested species, except for *Aedes aegypti*, for which the repellency ranged from 31.7% to 39.3%.

### *Pogostemon cablin* (Patchouli)

*Pogostemon cablin*, known as patchouli, is a perennial tropical herb with a long history of traditional use. It is widely used by the industry in the production of fragrances and cosmetics due to its pleasant and delicate aroma^21^.

In total, three studies reported the repellent efficacy of patchouli. Mustapa *et al*.^11^ observed that a 10% concentration of the essential oil provided 95% repellency for up to six hours. In the study by Deng *et al*.^22^, patchouli offered 55 min of protection against *Aedes aegypti*.

Sutthanont *et al*.^12^ found protection times of 60, 120, and 180 min using 100 µL of the undiluted essential oil against three culicid species: *Aedes aegypti, Anopheles dirus*, and *Culex quinquefasciatus*, respectively.

### Repellent testing methodology

The predominant method was the cage assay, which was used in 25 of the 33 reviewed studies^1,3-5,7,8,11-14,16,17,22-34^. This method is considered the "gold standard" for evaluating repellent activity in the laboratory, as per the World Health Organization^35^.

Other methodologies included the excito-repellency test system^19,20,36-39^, the Y-tube olfactometer^40^, and the Klun & Debboun test module^2^.

To ensure the uniformity of the animal model, all 33 studies standardized the baseline characteristics of the mosquitoes (such as age, sex, and fasting period), rearing conditions, temperature, and humidity.

### Risk of bias assessment

This review only included primary and experimental studies. Overall, most showed a low risk of bias.

### Selection bias

The assessment of selection bias aims to ensure that experimental groups (animals) were formed fairly, guaranteeing an undistorted outcome. Selection bias is evaluated across three domains (Table 2, columns 1, 2, and 3): sequence generation, baseline characteristics, and allocation concealment. Sequence generation describes the methods (if any) for random distribution to prevent manipulation. This review rated all evaluated studies as having an unclear risk of bias in this domain as they had no clear description of the order of mosquito collection or manner of group distribution.

**Table 2:** Assessment of bias risk in the included studies using SYRCLE

Article		Selection bias			Performance bias		Detection bias		Friction bias	Reporting bias	Others
		1	2	3	4	5	6	7	8	9	10
1.	Manh and Tuyet^5^	?	Y	N	?	N	Y	N	Y	Y	Y
2.	Boonyuan *et al*.^19^	?	Y	N	?	N	Y	N	Y	Y	Y
3.	Zhang *et al*.^1^	?	Y	N	?	N	Y	N	Y	Y	Y
4.	Fikru *et al*.^23^	?	Y	N	?	N	Y	N	Y	Y	Y
5.	Batume *et al*.^40^	?	Y	N	?	N	Y	N	Y	Y	Y
6.	Opoku-Bamfoh *et al*.^14^	?	Y	N	?	N	Y	N	Y	Y	Y
7.	De Brito *et al*.^7^	?	Y	N	?	N	Y	N	Y	Y	Y
8.	Haris *et al*.^13^	?	Y	N	?	N	Y	N	Y	Y	Y
9.	Haris *et al*.^24^	?	Y	N	?	N	Y	N	Y	Y	Y
10.	Dutta *et al*.^17^	?	Y	N	?	N	Y	N	Y	Y	Y
11.	Benelli *et al*.^25^	?	Y	N	?	N	Y	N	Y	Y	Y
12.	Parveen *et al*.^26^	?	Y	N	?	N	Y	N	Y	Y	Y
13.	Mustapa *et al*.^11^	?	Y	N	?	N	Y	N	Y	Y	Y
14.	Kamaraj *et al*.^8^	?	Y	N	?	N	Y	N	Y	Y	Y
15.	Deng *et al*.^22^	?	Y	N	?	N	Y	N	Y	Y	Y
16.	Tian *et al*.^27^	?	Y	N	?	N	Y	N	Y	Y	Y
17.	Najar *et al*.^28^	?	Y	N	?	N	Y	N	Y	Y	Y
18.	Abbas *et al*.^4^	?	Y	N	?	N	Y	N	Y	Y	Y
19.	Sheikh *et al*.^2^	?	Y	N	?	N	Y	N	Y	Y	Y
20.	Liakakou *et al*.^29^	?	Y	N	?	N	Y	N	Y	Y	Y
21.	Sutthanont *et al*.^12^	?	Y	N	?	N	Y	N	Y	Y	Y
22.	Ndirangu *et al*.^30^	?	Y	N	?	N	Y	N	Y	Y	Y
23.	Oliveira *et al*.^36^	?	Y	N	?	N	Y	N	Y	Y	Y
24.	Ludia *et al*.^3^	?	Y	N	?	N	Y	N	Y	Y	Y
25.	Iqbal *et al*.^16^	?	Y	N	?	N	Y	N	Y	Y	Y
26.	Osei-Owusu *et al*.^31^	?	Y	N	?	N	Y	N	Y	Y	Y
27.	Jhaiaun *et al*.^37^	?	Y	N	?	N	Y	N	Y	Y	Y
28.	Adhikari *et al*.^32^	?	Y	N	?	N	Y	N	Y	Y	Y
29.	Wahab *et al*.^38^	?	Y	N	?	N	Y	N	Y	Y	Y
30.	Silva *et al*.^39^	?	Y	N	?	N	Y	N	Y	Y	Y
31.	Abbas *et al*.^33^	?	Y	N	?	N	Y	N	Y	Y	Y
32.	Paulraj *et al*.^34^	?	Y	N	?	N	Y	N	Y	Y	Y
33.	Sukkanon *et al*.^20^	?	Y	N	?	N	Y	N	Y	Y	Y

Y = YES (low risk of bias); N = NO (high risk of bias);? (Unclear) = Risk of bias uncertain/nuclear; 1 = Sequence generation: in this domain, all studies failed to clearly specify how they generated sequences; 2 = Baseline characteristics: all studies standardized baseline characteristics such as species, age, and fasting of Culicidae; 3 = Allocation concealment: in this domain, no study reported concealment of treatments; 4 = Random housing: no study described the randomness of mosquito housing; 5 = Blinding: no study blinded applicators or volunteers; 6 = Evaluation of random outcomes: mosquitoes were distributed evenly in the cages, and the outcomes (landings/bites) were recorded; 7 = Blinding: no study blinded the evaluator to the outcome; 8 = Incomplete results: all studies reported complete results; 9 = Selective reporting of results: no studies omitted outcomes; 10 = Other sources of bias: no article showed other sources of bias.

The domain for baseline characteristics seeks to describe all possible factors or characteristics of the animals being compared to determine if the groups were similar at the start of the experiment. All reviewed articles showed a low risk of bias in this domain; that is, they described having standardized all baseline characteristics of their mosquito groups (e.g., age, sex, feeding time).

The allocation concealment domain refers to the methods to conceal the allocation sequence in sufficient detail to determine whether the allocations could have been foreseen during the experiment. Regarding this, all studies shows a high risk of bias as their applicators were aware of the allocation of mosquitoes to the treatments.

### Performance bias

Performance bias refers to any distortion that may occur in results whether by differences in how the groups were treated (administration of substances) or by their handling during the experiment. Performance bias is assessed across two domains (Table 2, columns 4 and 5): random housing and blinding.

Random housing examines how the animals were allocated within the experimental environment to ensure that no group was favored. This review classified all studies as having an unclear risk of bias in this domain as they lacked information on how the mosquitoes were distributed in the cages.

The blinding domain aims to describe what measures were taken to blind the caregivers or researchers in the study to prevent them from knowing which intervention each animal would receive. No study mentioned any attempt to blind their applicators; thus, all were aware of the intervention (application of the substance and the control), which suggests a high risk of bias.

### Detection bias

Detection bias seeks to elucidate the effects of the intervention related to factors that could influence outcome measurement or description. It is assessed across two domains (Table 2, columns 6 and 7): random outcome assessment and blinding.

In this review, random outcome assessment verifies whether the mosquitoes were randomly selected for outcome evaluation (e.g., protection times, number of landings, bites, and escape rate). Regarding, all studies indicated a low risk of bias as they systematically assessed mosquitoes, showing organized monitoring.

The blinding domain describes all measures to blind outcome assessors, preventing them from knowing which treatment each animal would receive. In this domain, all studies showed a high risk of bias, suggesting that the assessors were aware of the applied treatment.

### Attrition bias

Attrition bias occurs when there exists a risk of distortion in the results due to the loss of data throughout the experiment. This bias is assessed by one domain (Table 2, column 8): incomplete outcome data.

The "incomplete outcome data" domain assesses data completeness and whether the studies included all results in their outcomes. This review determined that the chosen studies included all results in their reported outcomes. Therefore, it classified all studies as having a low risk of bias in this domain.

### Reporting bias

Reporting bias (Table 2, column 9) occurs when results are reported selectively; that is, only some outcomes are reported, whereas others are omitted. This bias is assessed by one domain: selective outcome reporting. This domain describes examined and found instances of selective reporting.

The selected studies reported all their outcomes (described in the methods as the number of landings and bites in the treatment and control groups) in their results with no apparent omission.

### Other bias

Other sources of bias (Table 2, column 10, "other" domain) serve as an open category to check for any type of bias unidentified in the other domains. None of the studies analyzed showed other sources of bias, suggesting a low risk of bias for these studies in this domain.

## DISCUSSION

### Evidence of efficacy and mechanism of action

Repellents based on natural products are considered promising alternatives against culicids due to their safety and efficacy in various studies^41^. As evinced in this review, plants such as *Pogostemon cablin* (patchouli)^11,12,21,22^, *Cananga odorata* ^18-20^, *Citrus* spp.,^4,8,12,17^ *Mentha* spp. (mint)^5,8,13,16^, *Ocimum basilicum* (basil)^3,12,14^ *Eucalyptus* spp.^2,8,13^ and *Cymbopogon* spp. (citronella)^7,8,11,12^ showed significant repellent activity, with protection percentages varying according to the concentration and the targeted mosquito species.

Over the years, plants have developed a wide variety of morphological and chemical defense strategies to reduce herbivory by insects^42^. These defense mechanisms fall into two categories: constitutive and induced. Constitutive defenses are always present (e.g., physical barriers), even in the absence of attacks. On the other hand, induced defenses are activated when the plant is under attack, primarily consisting of chemical responses^43,44^.

Regarding repellent effect specificity, the results indicate that efficacy varies across mosquito genera. For example, although *Cymbopogon* oils have shown high initial repellency against *Aedes aegypti*, their performance against *Culex quinquefasciatus* was often slightly lower regarding protection time^6,11^. Moreover, *eucalyptus* species performed remarkably well against *Anopheles* spp. mosquitoes, often with longer protection times than other botanical species^12^. These differences are likely due to the olfactory receptors and behavioral patterns of each genus, suggesting that the development of natural repellents should consider target species to optimize protection.

Essential oils stand out among chemical defenses. These complex mixtures of secondary metabolites had several ecological functions^45^. Approximately 100,000 secondary metabolites are produced by several metabolic pathways^46^. These compounds generally fall into four main categories: phenolics, terpenes, and nitrogen and sulfur containing metabolites^47^.

In this context, some plants are widely exploited for their repellent properties, such as *Cymbopogon* spp. species, which commonly serve as protection against Culicidae. The essential oils from these plants contain compounds such as citronellal, ledol, α-pinene, β-pinene, and others^2^, which can act at the receptor level by interfering with the sensory perception of mosquitoes.

Du *et al*.^48^ showed that citronellal derivatives can directly potentiate the TRPA1 gene antennal olfactory receptor in *Anopheles gambiae*. Another study found that citronella directly activates cation channels, being compared to the excito-repellent pyrethrin, another plant-derived repellent^49^. However, this mechanism contradicts the inhibitory influence exerted by DEET^50^. Although the protection offered by citronella is inferior to that of DEET, it still provides sufficient protection against culicids. For other plants, the underlying mechanisms require further explanation.

Formulations such as nanoemulsions can be developed to increase the efficacy of essential oils, prolonging their longevity and promoting controlled release. Studies have shown that a nanoemulsion of citronella essential oil showed high residual activity, maintaining its efficacy for up to 21 days^51^. Furthermore, nanoemulsions of *Eucalyptus globulus* and *Mentha piperita* significantly increased the bioavailability of these oils in the environment^52^.

### Repellency methodologies and influencing factors

As previously described, cage assays were the predominant method in the reviewed studies. Recommended by the World Health Organization^35^, this assay applies the tested product to a delimited area on a volunteer's forearm, who then inserts their limb into a cage for a predetermined amount of time.

Interpreting the results of these assays requires caution. The first aspect to consider is the effect of perspiration on the efficacy of the repellent in cage assays, especially concerning the protection time. Rodriguez *et al*.^53^ highlight that, to achieve durability, repellents need to adhere via substantivity (non-penetrating adherence to the superficial layers of the skin), which reduces its loss by perspiration. Furthermore, different skin types may absorb the repellent at varying levels, which can also influence effectiveness^54^.

The second aspect to consider is that prolonged stress can influence the non-feeding behavior of mosquitoes in cage assays. Fradin *et al*.^55^ described that continuous exposure to the repellent in cage assays can fatigue the mosquitoes or prolong the blockage of their antennal chemoreceptors.

The third aspect to be considered concerns the possibility of skin irritation caused by some essential oils. Therefore, plants with irritant properties must be evaluated beforehand to ensure their safe use in future repellent formulations^56^.

The excito-repellency system evaluates treatments by measuring the escape of mosquitoes from a treated chamber to an untreated one^57^. Its evaluation has weaknesses: the complexity of the test and the impracticality of its execution in certain laboratories as not all possess the necessary infrastructure. Another critical point is that the treatment is not applied to human skin, which may prevent the results from reflecting the real-world application of a repellent. This necessitates additional tests^58^.

In contrast, the Y-tube olfactometer assay measures the spatial action of a treatment by evaluating the number of mosquitoes that fly toward it when compared to a control path^59^. Such assays commonly serve to study how arthropods locate their hosts^60^. According to Luker^58^, this assay model has strengths, such as being accessible, easy to implement, and practical for most laboratories. One of its limitations refers to its inability to test contact repellency, which requires additional tests for support.

### Bias assessment and recommendations

Bias analysis must consider potential biases associated with human volunteers, a domain ignored by SYRCLE. Factors such as the lack of blinding of participants and assessors regarding the application of repellents can induce performance and detection biases. Furthermore, the selection of volunteers and the absence of reports on their individual characteristics (e.g., diet or skin metabolites)^53^, which are associated with mosquito attractiveness, may introduce a potential selection bias. Although SYRCLE can robustly evaluate animal models, this review recommends that future studies apply a specific tool for human studies (ROBINS-I) to ensure the validity of individual protection results.

The chosen studies evaluated repellent activity against various Culicidae species, such as *Aedes* spp., *Culex* spp., and *Anopheles* spp^2,8,22,25,27^.

Although methodological variability between studies poses a challenge, this review found plant species, such as *Cymbopogon spp*., and *Eucalyptus spp*., with consistent evidence of efficacy, providing a solid basis for candidates for the development of commercial repellents.

This review found heterogeneous results that vary regarding compound concentrations, dosage, mosquito species, and repellency assessment^2,19,36,37^. In this review, most studies (78.8%, n=26) described protection time and repellency percentage. However, to ensure a comprehensive analysis, we also included studies that only reported one of these metrics (21.2%, n=7); some focused on protection time^1,5,7,13,14,23,24,40^ and others, on efficacy percentages^2,3,16,20,31,32,37-39^.

The number of human volunteers varied between studies, ranging from 1 to 15 individuals per trial^2,10,34^. Similarly, the density and species of mosquitoes in the tests showed great heterogeneity, with some studies focusing on a single species, such as *Aedes aegypti*, whereas others tested the oils against up to four species (e.g., *Aedes, Anopheles*, and *Culex*)^1,5,11,14,29^.

Future research should clearly define the used repellents, volunteers’ characteristics, the tested mosquito species, and the evaluated parameters. These studies must consider protection time and efficacy percentage to more comprehensively assess repellent activity. Moreover, it is important to emphasize that the studies in this review were conducted under controlled laboratory conditions. Although laboratory tests provide essential standardized data, they fail to fully replicate environmental variables such as wind, humidity, temperature, and host-seeking behavior in nature. Therefore, future research should prioritize field tests to validate the efficacy of these essential oils, a crucial step in the development of commercial biorepellents.

To improve the reproducibility and comparability of results related to essential oils as biorepellents, subsequent investigations should follow standardized experimental and reporting protocols. We recommend that future studies standardize concentration units (preferably using mg/cm^2^ or absolute concentration) to avoid the ambiguity of percentages or volume/volume ratios that depend on the initial applied amount; harmonize methodologies and follow World Health Organization or ASTM International guidelines for repellent testing to ensure consistent exposure conditions; and detail the recruitment and preparation of volunteers, specifying a minimum sample size to ensure statistical power and describing pre-test restrictions (e.g., avoiding scented soaps, alcohol, or nicotine) that may interfere with mosquito attraction.

## CONCLUSION

The data show that essential oils offer promising natural alternatives for mosquito protection, exhibiting significant repellent activity against Culicidae. While certain botanical families provide immediate repellency, others stand out for the longevity of their protection, with efficacy often specific to the genus. However, the transition from laboratory success to commercial application faces challenges due to the lack of standardized experimental protocols and the predominance of studies under controlled conditions rather than field trials. Future research should prioritize methodological harmonization and clinical validation in natural environments. These findings reinforce the potential of essential oils as key components in integrated vector management and the development of sustainable biorepellents.

## Data Availability

The complete anonymized dataset supporting the findings of this study is included within the article.
